# Modulating Effects of the Hydroethanolic Leaf Extract of *Persicaria lanigera* R. Br. Soják (Polygonaceae) against Acute Inflammation

**DOI:** 10.1155/2023/5567410

**Published:** 2023-07-10

**Authors:** Meshack Antwi-Adjei, Ernest Obese, Emmanuel Awiintig Adakudugu, Isaac Tabiri Henneh, Robert Peter Biney, Benjamin Aboagye, Benjamin Amoani, Daniel Anokwah, Elvis Ofori Ameyaw

**Affiliations:** ^1^Department of Pharmacology, School of Medical Sciences, University of Cape Coast, Cape Coast, Ghana; ^2^Department of Pharmacotherapeutics and Pharmacy Practice, School of Pharmacy and Pharmaceutical Sciences, University of Cape Coast, Cape Coast, Ghana; ^3^Department of Forensic Sciences, School of Biological Sciences, University of Cape Coast, Cape Coast, Ghana; ^4^Department of Biomedical Sciences, School of Allied Health Sciences, University of Cape Coast, Cape Coast, Ghana; ^5^Department of Pharmacognosy and Herbal Medicine, School of Pharmacy and Pharmaceutical Sciences, University of Cape Coast, Cape Coast, Ghana

## Abstract

Plant species have been used traditionally to treat numerous inflammatory disorders because of their known medicinal properties. This study aimed to assess the anti-inflammatory effect of aqueous ethanolic leaf extract of *Persicaria lanigera* using acute inflammatory models. The safety profile of the *Persicaria lanigera* extract was assessed using an acute toxicity model. The anti-inflammatory effect of the *Persicaria lanigera* leaf extract (100–600 mg·kg^−1^, p.o.) was studied in carrageenan-induced paw oedema, zymosan-induced knee joint arthritis, and histamine-induced paw oedema in Sprague–Dawley rats (*n* = 5). It was observed that the *Persicaria lanigera* leaf extract administered prophylactically significantly inhibited paw oedema from 99.01 ± 12.59 to 59.10 ± 4.94%, 56.08 ± 3.65%, and 48.62 ± 3.27% at 100 mg·kg^−1^, 300 mg·kg^−1^, and 600 mg·kg^−1^, while the standard drug, aspirin, showed 41.84 ± 9.25% in carrageenan-induced paw oedema, respectively. Furthermore, the extract decreased knee joint inflammation significantly from 62.43 ± 5.73% to 32.07 ± 2.98% and 24.33 ± 8.58% at 300 mg·kg^−1^ and 600 mg·kg^−1^ in zymosan-induced knee joint inflammation, respectively. In the histamine-induced paw oedema model, the extract significantly inhibited oedema to 61.53 ± 9.17%, 54.21 ± 9.38%, and 54.22 ± 9.37% at the same doses. Aqueous ethanolic leaf extract of *Persicaria lanigera* is safe and attenuates inflammation in acute inflammation models.

## 1. Introduction

Inflammation is defined as the body's reaction to damaging stimuli such as infections, toxins, or heat, as well as autoimmune illnesses [1]. This is the body's major method for tissue repair following injury, and it entails a sequence of cellular and vascular processes that culminate in the removal of damaged tissues and the regeneration of new ones. Increased vascular permeability, cellular adhesion, cell migration, and oedema production are all part of this chain of events [2]. Although inflammation is supposed to be self-regulating, a persistently unregulated inflammatory response can lead to chronic inflammatory conditions, such as rheumatoid arthritis, gastrointestinal diseases, asthma, or autoimmune disorders, and, as a result, function loss [3, 4]. Currently, inflammatory diseases are managed with orthodox drugs such as steroidal anti-inflammatory drugs, nonsteroidal anti-inflammatory drugs (NSAIDs), and disease-modifying antirheumatoid drugs (DMARDs) [5, 6]. Usually, the use of these medications is associated with numerous adverse effects including obesity, dyspepsia, gastric ulcers, renal damage, and immunosuppression [7, 8].

Medicinal plant species are reported to play a major role in drug discovery and have become essential sources with pharmacological potential for the development of safe and potent drugs [9].

Therefore, the search for new drugs with no or fewer adverse effects for the management of inflammatory diseases is very crucial. *Persicaria lanigera* is a medicinal plant that can be explored to mitigate inflammatory conditions. It belongs to the family Polygonaceae which can be located in tropical and subtropical regions [10]. The Polygonaceae family has been linked to a variety of medical uses, including the treatment of ulcerative colitis, intestinal parasites, asthma, bronchitis, inflammatory disorders, and diarrhea [11]. Obese and his colleagues (2021) demonstrated the analgesic properties of this plant; however, there is no scientific report on its anti-inflammatory activities to support its folkloric use in the management of inflammatory diseases. Hence, this study aimed to evaluate the anti-inflammatory effect of the aqueous ethanolic leaf *Persicaria lanigera* extract on inflammatory models in Sprague–Dawley rats.

## 2. Materials and Methods

### 2.1. Materials

#### 2.1.1. Plant Collection and Extraction

The leaves of *Persicaria lanigera* were collected in Effutu along the Jukwa road in Cape Coast, Central Region, between September and November 2018. The plant was identified and authenticated subsequently by Mr. Felix Fynn, a botanist at the School of Biological Sciences Herbarium, University of Cape Coast (UCC), and a voucher specimen (no. MAA003) was kept in the herbarium of the School of Biological Sciences, UCC.

To prepare the aqueous ethanolic extract, the leaves of the plant were air-dried at room temperature for 21 days and later pulverized with a heavy-duty blender (3628GL72- 430CB2- Waring, USA) into fine powder. Afterwards, 600 g of the powder was extracted by cold maceration using 2.0 L of 70% ethanol for 72 h and the resulting supernatant was decanted and filtered using a Buchner funnel and Whatman filter paper no. 1. The filtrate was then concentrated under a reduced pressure at a low temperature of 50°C using a rotary evaporator (Model: R-290, BUCHI, Switzerland) to obtain dark-green liquid which was evaporated to dryness in an oven (Gallenkamp OMT oven, Sanyo, Japan) at 50°C for 24 h. Later, the semisolid dried extract was kept in a refrigerator (LG Haiser 220L Freezer, Ningbo Haiser Electronic Appliance Co., Ltd., Zhejiang, China) until use. A final yield of 9.3% (*w*/*w*) was obtained. The extract was referred to as *Persicaria lanigera* extract (PLE) and kept in a desiccator.

#### 2.1.2. Animals

Sprague–Dawley rats (200–250 g) of both sexes were purchased from the Center for Plant Medicine Research, Mampong-Akwapim, Ghana, and housed in the animal facility of the Department of Biomedical Sciences, UCC. Following the Animal Welfare Regulations and the Public Health Service Policy on Humane Care and Use of Laboratory Animals (PHS 2002), all animals used in this study were humanely handled throughout the experimental period. Moreover, studies on rodents were conducted with the approval of the Research and Ethics Committee of the School of Pharmacy and Pharmaceutical Sciences, University of Cape Coast (approval number UCCSoPPS/REC/19/008). The animals were randomly grouped (*n* = 5) and housed in stainless steel cages (34 cm × 47 cm × 18 cm) with softwood shavings as bedding and were fed with a normal commercial pellet diet (Agricare, Tema, Ghana). All animals were given access to water *ad libitum*. The animals were allowed enough time to acclimatize to the new environment and were maintained at a room temperature of 26 ± 2°C in a 12 h light-dark cycle. Each animal was used only once, and at the end of each experiment, all animals were euthanized.

#### 2.1.3. Chemicals and Reagents

Carrageenan sodium salt (98% purity) (Sigma-Aldrich, St Louis, MO, USA), zymosan A (98% purity) (Carbosynth Ltd., Compton, United Kingdom (UK)), aspirin tablets (98% purity) (MP-Biomedicals, California, USA), acetic acid (99.5% purity) (Ernest Chemist, Accra, Ghana), sulphasalazine (98% purity) (Shire Pharmaceuticals Inc., MA, USA), ethanol (95% purity) (Ernest Chemist, Accra, Ghana), and histamine powder (99% purity) (Sigma-Aldrich, St Louis, MO, USA) were used.

### 2.2. Methods

#### 2.2.1. Acute Toxicity Studies of *Persicaria lanigera*

An acute toxicity study of PLE was performed as per the procedure described by the Organisation for Economic Co-operation and Development (OECD) guidelines-425 [12, 13]. Sprague–Dawley rats (100–150 g, *n* = 5) of both sexes were randomly selected and divided into six (6) groups. Animals were allowed to acclimatize to the laboratory environment for 1 week and fasted overnight with access to water *ad libitum*. Animals were weighed before oral administration of PLE in doses of 100 mg·kg^−1^ (Group II), 300 mg·kg^−1^ (Group III), 1000 mg·kg^−1^ (Group IV), 3000 mg·kg^−1^ (Group V), and 5000 mg·kg^−1^ (Group VI). The control group (Group I) received 10 ml·kg^−1^ of distilled water. Individual animals in all treatment groups were critically observed for a period of 24 h for general behaviour changes, alterations in physiological function, and mortality. Animals were monitored at 0, 15, 30, 60, 120, 180, 240 min, and 24 h for convulsions, tremors, excitement, respiratory changes, unusual locomotion, agitation, ataxia, aggression, sedation, salivation, urination, and defaecation after oral PLE (100–5000 mg·kg^−1^) administration. Using the Basante-Romo et al. (2021) scaling method, observations made for all toxicity signs were scored on a scale of 0–3 [14]. The lethal dose (LD_50_) of PLE was estimated using the following equation [15]:

(1) $$\begin{array}{c} {LD}_{50}=\sqrt{\frac{D_{0}+D_{100}}{2}}\text{,} \end{array}$$

where *D*_*o*_ is the maximum dose that resulted in 0% mortality; *D*_100_ is the minimum dose that resulted in mortality.

#### 2.2.2. Carrageenan-Induced Paw Oedema in Rats

Paw oedema was induced by subplantar injection of 0.1 ml of 1% (*w*/*v*) sterile carrageenan (dissolved in 0.9% saline solution) into the right hind limbs of rats (100–150 g, *n* = 5) [16, 17]. The initial basal thickness of the animals was measured using an electronic digital Vernier caliper (VC1346i, MP Lab, Equip, USA) before the carrageenan injection. Paw thickness of the injected limb was then measured at an hourly interval for 5 h. Inhibition of oedema was expressed using the following equation:

(2) $$\begin{array}{c} \%\;change\;in\;paw\;thickness=\left(\frac{\left(P_{t}-P_{o}\right)}{P_{o}}\right)\times 100\text{,} \end{array}$$

where *P*_*o*_ is the paw thickness before carrageenan injection (i.e., time zero). *P*_*t*_ is the paw thickness (at various time intervals) after carrageenan injection.

The percentage change in paw thickness for each animal was calculated from the raw scores at time 0 and then averaged. Total oedema was expressed as the area under the time-course curve (AUC), and the percentage inhibition of oedema was calculated using the following equation:

(3) $$\begin{array}{c} \%\;inhibition\;of\;oedema=\left(\frac{{AUC}_{\left(control\right)}-{AUC}_{\left(treatment\right)}}{{AUC}_{\left(control\right)}}\right)\times 100\text{.} \end{array}$$

In the prophylactic study, PLE (100, 300, 600 mg·kg^−1^, p.o) or aspirin (100 mg·kg^−1^, p.o) were administered before carrageenan inoculation. In the therapeutic study, drugs were administered 1 h after subplantar injection of carrageenan solution. The control rats orally received distilled water (10 ml·kg^−1^).

#### 2.2.3. Zymosan-Induced Acute Knee Arthritis in Rats

*(1) Induction of Acute Arthritis and Evaluation of Knee Joint Inflammation*. Using a method earlier described by Mortada and Hussain (2014) [18], Sprague–Dawley rats (100–150 g, *n* = 5) were randomly selected into six (6) groups (I–VI) and joint inflammation was induced by injecting 500 *µ*g of zymosan in 25 *µ*l of normal saline in each knee joint cavity of the right limb. Before induction, hair around the knee joint was carefully shaved to expose the joint and cleaned with 70% alcohol. The initial right knee joint thickness (transverse diameter, mm) of each rat in all groups (I–VI) was measured using a digital caliper (VC1346i, MP Lab Equip, USA) and recorded.

  Group I (naïve): received only 25 *µ*l of paraffin oil
  Group II (arthritic): received only 500 *µ*g of zymosan in 25 *µ*l of saline
  Group III: received 100 mg·kg^−1^ of aspirin (p.o) + 500 *µ*g of zymosan in 25 *µ*l of saline
  Group IV: treated with PLE (100 mg·kg^−1^, p.o)
  Group V: treated with PLE (300 mg·kg^−1^, p.o)
  Group VI: treated with PLE (600 mg·kg^−1^, p.o)

Knee joint swelling was assessed by measuring the knee joint of each rat in all treatment groups at an hourly interval of 5 h. Inhibition of joint swelling was determined using the procedure described in Section 2.2.2.

*(2) Determination of Joint Neutrophils and Leukocyte Infiltration*. Neutrophil and leucocyte infiltration into the knee joint cavity was determined after 5 h of intra-articular injection of zymosan. Under light ether anaesthesia, blood samples were collected from each knee joint synovial cavity into EDTA tubes. Blood samples were analyzed for neutrophils and total leucocytes using an automated haematology cell diagnosis analyzer (HP-HEMA6500A, Zhengzhou Hepo International Trading Co. Ltd., Henan, China).

*(3) Histopathological Evaluation of the Knee Joint*. Knee joint tissues were removed from each rat in the various treatment groups to assess the histopathology of the tissue cavity after arthritic induction. In brief, formalin-fixed tissues were decalcified, dehydrated, embedded in paraffin oil, and sectioned for histopathological studies. The prepared sections were stained with hematoxylin and eosin (H&E), and histological alterations were examined by light microscopy using a microscope (BS-2040 fb LED, Movel Scientific Instrument Co. Ltd., Zhejiang, China).

*(4) Determination of Mast Cell Proliferation*. Thick segmented sections of the knee joint tissues were fixed in Carnoy's fixative and stained with 1% toluidine blue. Using a micrometer grid (0.042 mm^2^), mast cells were counted in coded sections at ×20 magnification.

#### 2.2.4. Histamine-Induced Paw Oedema

Paw oedema was induced by injecting 0.1 ml of 1% histamine (freshly prepared in normal saline) into the subplantar tissues of the right hind paw of rats [17]. In brief, Sprague–Dawley rats (100–150 g, *n* = 5) of both sexes were allowed to fast overnight and grouped into five (5) groups. The initial paw thickness of both limbs of rats was measured before oedema induction. Paw thickness was measured at 30, 60, 90, 120, and 180 min using a Vernier caliper (VC1346i, MP Lab, Equip, USA). Drugs were administered 60 min before histamine injection.

  Group I (negative control): received 1 ml of normal saline
  Group II (positive control): received 4 mg·kg^−1^ of chlorpheniramine (dissolved in saline, p.o)
  Group III: received PLE (100–600 mg·kg^−1^, p.o), respectively
  Group IV: received PLE (300 mg·kg^−1^, p.o)
  Group V: received PLE (600 mg·kg^−1^, p.o)

Percentage changes in paw thickness and total paw oedema were determined using the formula stated above (in Section 2.2.2).

## 3. Data Analysis

All data were presented as the mean ± SEM (*n* = 5) of the effect of drugs on the maximal and total oedema effects over the period. Data were analyzed statistically through a test of significance using both one-way and two-way analysis of variance (ANOVA) followed by Dunnet's *post hoc* test. All graphs were plotted using GraphPad Prism version 7.0 (GraphPad, San Diego, USA).

## 4. Results and Discussion

### 4.1. Results

#### 4.1.1. Acute Toxicity Profile

Preliminary acute toxicity evaluation of PLE at doses of 100, 300, 1000, 3000, and 5000 mg·kg^−1^ showed no critical adverse effects that could lead to death, which is suggestive that the LD_50_ value of PLE could exceed 5000 mg·kg^−1^, thus indicating that the extract is nontoxic. Therefore, PLE (100–5000 mg·kg^−1^, p.o) did not cause any noticeable behavioural, physiological, or clinical signs that may lead to death, and thus, PLE was acutely safe (Table 1). However, 8 h after PLE administration, there were only mild signs of urination and defaecation observed in rats of PLE-treated groups of 3000 and 5000 mg·kg^−1^ (Table 1), but these signs were off gradually until the 24^th^ h. Hence, no mortality was recorded during the observation period in all PLE-treated groups (Table 1). The doses used in the anti-inflammatory activity test of PLE were based on the LD_50_ value.

#### 4.1.2. Carrageenan-Induced Paw Oedema in Rats

**Figure 1:**
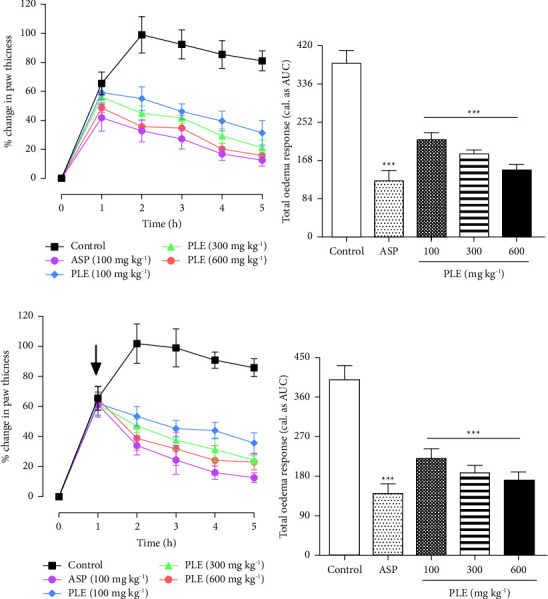
Effect of PLE on carrageenan-induced paw oedema in rats. The percentage change in paw oedema is shown in the time-course curve (a, c). Total paw oedema was determined as AUC (b, d), and data were presented as the mean ± SEM (*n* = 5). ^*∗∗∗*^*P* < 0.001 compared to the inflamed control response (two-way ANOVA followed by Dunnet's *post hoc* test). The arrow denotes the time of drug administration.

In the study, carrageenan injected into the right hind limbs of rats caused oedema of the limbs peaking between 2 and 3 h in the rats. In the prophylactic study, the percentage mean maximal oedema formed in control rats was 99.01 ± 12.59% (Figure 1(a)). Aspirin (100 mg·kg^−1^, p.o) significantly reduced the mean maximal oedema to 41.84 ± 9.25% (Figure 1(a)). PLE (100–600 mg·kg^−1^, p.o) administered preemptively similarly decreased the mean maximal swelling to 59.10 ± 4.94%, 56.08 ± 3.65%, and 48.62 ± 3.27% at 100, 300, and 600 mg·kg^−1^ when compared to the control group, respectively (Figure 1(a)). The total paw oedema formed after 5 h of oedema induction was significantly (*P* < 0.001) reduced by 43.72%, 52.34%, and 61.58% at 100, 300, and 600 mg·kg^−1^ relative to the control response, respectively (Figure 1(b)).

In the therapeutic study, either aspirin (100 mg·kg^−1^) or PLE (100–600 mg·kg^−1^) was administered after (30 min) carrageenan injection, and the mean maximal swelling attained by the control group was 101.86 ± 13.1% (Figure 1(c)). Aspirin (100 mg·kg^−1^, p.o) suppressed the mean maximal oedema formed significantly to 61.29 ± 8.43% when compared to the control group (Figure 1(c)). Similarly, PLE (100–600 mg·kg^−1^, p.o) significantly suppressed the mean maximal oedema formed to 61.97 ± 7.75%, 61.42 ± 7.59%, and 65.24 ± 8.07% at 100, 300, and 600 mg·kg^−1^ when compared to the control group, respectively (Figure 1(c)). The total paw oedema attained was significantly (*P* < 0.001) reduced by 43.72%, 52.3%, and 61.58% at 100, 300, and 600 mg·kg^−1^ when compared to the control response, respectively (Figure 1(d)).

#### 4.1.3. Zymosan-Induced Acute Knee Joint Arthritis in Rats

**Figure 2:**
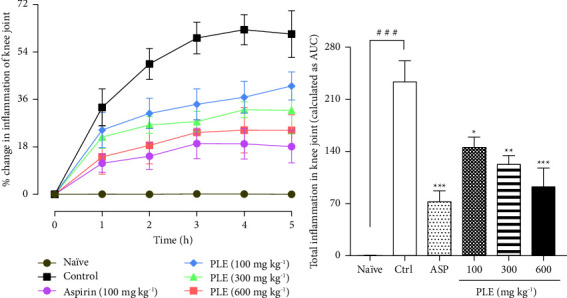
Effect of PLE on zymosan-induced acute knee joint arthritis in rats. Joint swelling was observed at 1 h intervals for 5 h as a percentage change in paw thickness (a). Total paw oedema was determined as AUC (b) and data were presented as the mean ± SEM (*n* = 5). ^###^*P* < 0.001; ^*∗*^*P* < 0.05; ^*∗∗*^*P* < 0.01; ^*∗∗∗*^*P* < 0.001 compared to the inflamed control response (two-way ANOVA followed by Dunnet's *post hoc* test).

*(1) Knee Joint Inflammation Assessment*. In this study, the induction of acute joint inflammation as a result of intra-articular injection of zymosan caused swelling of the knee joint. In the study, the naïve (paraffin oil-treated) group showed no swelling of the knee joint when compared to the control (zymosan-treated) group (Figure 2). However, there was severe swelling of the knee joints of the right limbs of rats in the negative control group that attained a mean maximal knee joint thickness of 62.43 ± 5.73% (Figure 2(a)). Aspirin (100 mg·kg^−1^, p.o) significantly decreased the mean maximal knee joint swelling attained to 19.28 ± 5.78% relative to the control group (Figure 2(a)). Similarly, PLE (100–600 mg·kg^−1^, p.o) showed a significant reduction of the mean maximal knee joint thickness attained to 32.07 ± 2.98% and 24.33 ± 8.58% at 300 and 600 mg·kg^−1^ when compared to the control response, respectively (Figure 2(a)). The total knee joint swelling attained after 5 h of acute knee joint arthritis induction was suppressed significantly by 37.51%, 47.27%, and 60.46% at 100, 300, and 600 mg·kg^−1^ relative to the control response in a dose-dependent manner, respectively (Figure 2(b)).

**Figure 3:**
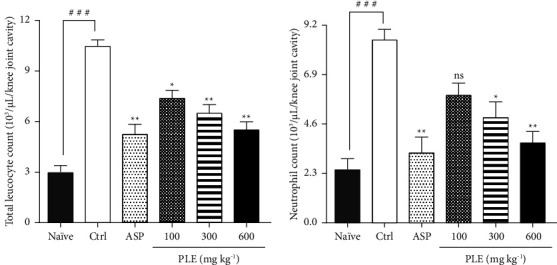
Effect of PLE on neutrophil (a) and total leukocyte (b) infiltration of the knee joint cavity in rats with zymosan-induced acute knee joint arthritis. Data were presented as the mean ± SEM. ^###^*P* < 0.001; ^*∗*^*P* < 0.05; ^*∗∗*^*P* < 0.01 when compared to the control group.

*(2) Neutrophil and Leucocyte Infiltration in the Knee Joint*. In the study, there was elevated infiltration of neutrophil and leucocyte levels in the knee joint cavity of the control group. The influx of neutrophils in the synovial joint of the knee increased to 8.51 ± 0.50 in the control group (Figure 3(a)). Aspirin (100 mg·kg^−1^, p.o) significantly inhibited the neutrophil infiltration in the knee cavity to 3.28 ± 0.73 when compared to the control group (Figure 3(a)). PLE (100–600 mg·kg^−1^, p.o) similarly inhibited the neutrophil influx in the knee cavity significantly to 4.93 ± 0.71 and 3.72 ± 0.54 at 300 and 600 mg·kg^−1^ when compared to the control group, respectively (Figure 3(a)). Total leucocyte infiltration into the knee cavity increased enormously to 10.46 ± 0.39 in the control group (Figure 3(b)). Aspirin (100 mg·kg^−1^, p.o) significantly inhibited the total leucocyte infiltration to 5.29 ± 0.60 when compared to the control group (Figure 3(b)). Similarly, PLE (100–600 mg·kg^−1^, p.o) showed a significant inhibition of total leucocyte migration into the knee joint cavity to 7.42 ± 0.47, 6.50 ± 0.51, and 5.51 ± 0.49 at 100, 300, and 600 mg·kg^−1^ when compared to the control group, respectively (Figure 3(b)).

**Figure 4:**
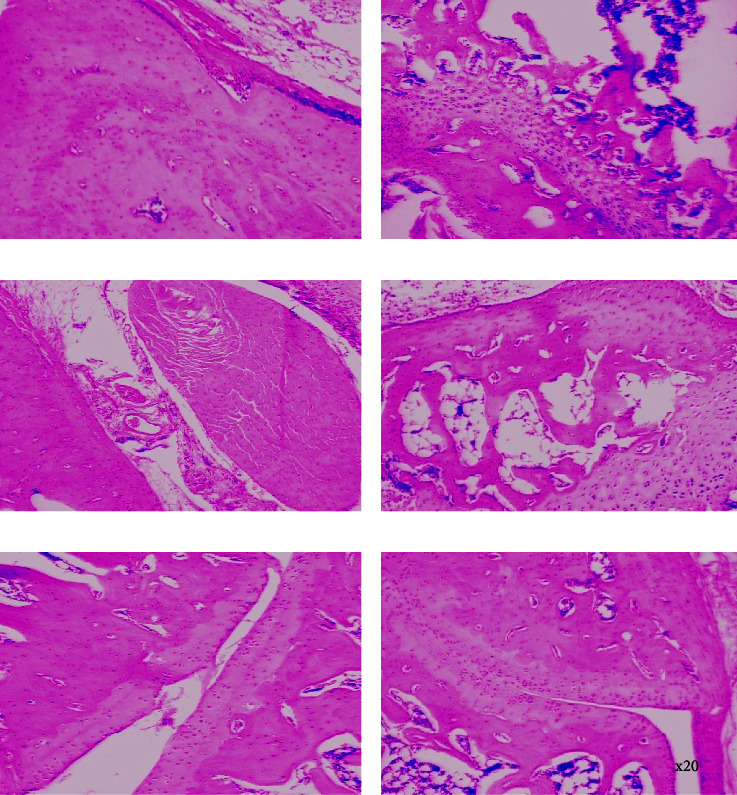
Histopathological assessment of PLE on zymosan-induced acute knee joint arthritis in Sprague–Dawley rats. Sections were made from the knee joint of the right hind limbs and stained using H&E stain. Naïve control (a), zymosan-treated (acute knee joint arthritic) control (b), aspirin 100 mg·kg^−1^ (c), PLE 100 mg·kg^−1^ (d), PLE 300 mg·kg^−1^ (e), and PLE 600 mg·kg^−1^ (f), respectively. The arrow indicates the degree of synovitis in the knee joint cavity.

*(3) Histopathological Evaluation of Knee Joint*. In the study, the naïve (paraffin-treated) group showed normal articular cartilage, bone, and synovium with no signs of pathological arthritis when compared to the control (zymosan-treated) group (Figure 4(a)). The zymosan-treated (negative control) group exhibited severe disruption of synovial membranes and bone cortex indicative of pathological arthritis characterized by marked increased inflammatory cell infiltration, decreased chondrocytes, and damaged cartilage (Figure 4(b)). However, aspirin (100 mg·kg^−1^, p.o) attenuated pathological arthritis by reducing cartilage and bone destruction, inflammatory cell infiltration, and synovium hypertrophy relative to the control group (Figure 4(c)). Similarly, PLE (100–600 mg·kg^−1^, p.o) ameliorated the histological changes associated with pathological arthritis by decreasing synovium hypertrophy, inflammatory cell infiltration, and improving knee joint and cartilage architecture at all doses relative to the control group (Figures 4(d)–4(f)).

**Figure 5:**
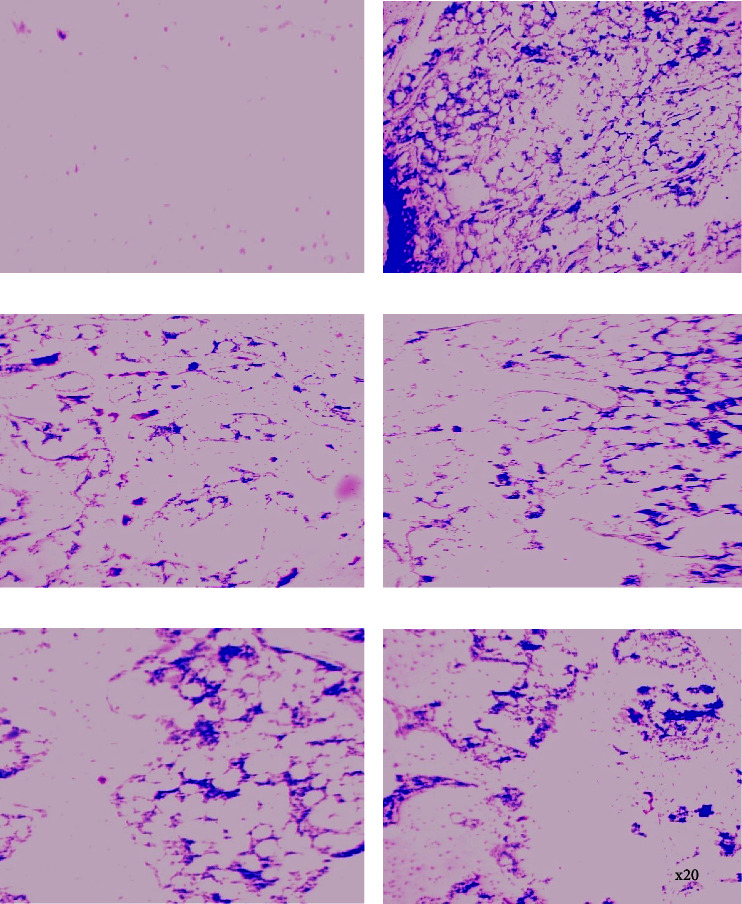
Mast cell proliferation in zymosan-induced acute knee joint arthritis in Sprague–Dawley rats. Naïve control (a), zymosan-treated (acute knee joint arthritic) control (b), aspirin 100 mg·kg^−1^ (c), PLE 100 mg·kg^−1^ (d), PLE 300 mg·kg^−1^ (e), and PLE 600 mg·kg^−1^ (f), respectively. The arrow shows mast cells.

**Figure 6:**
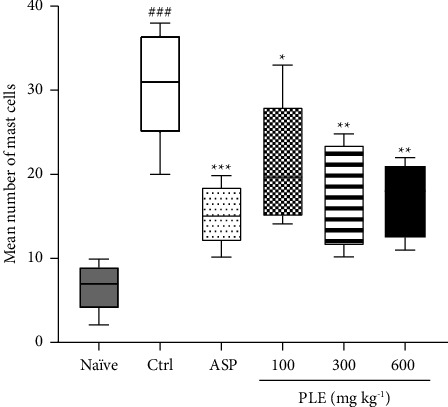
Effect of PLE on mast cell proliferation in zymosan-induced acute knee joint arthritis in Sprague–Dawley rats. Data are presented as the mean ± SEM (*n* = 5). ^###^*P* < 0.001; ^*∗*^*P* < 0.05; ^*∗∗*^*P* < 0.01; ^*∗∗∗*^*P* < 0.001 compared to the knee joint arthritic (zymosan-treated) control group (two-way ANOVA followed by Dunnet's *post hoc* test).

*(4) Mast Cell Proliferation in the Knee Joint Cavity*. In this study, the naïve (paraffin oil-treated) control showed no increased influx of mast cells to the joint tissues (Figure 5(a)) and significantly (*P* < 0.05) recorded a baseline mean total number of mast cells of 6.60 ± 1.33 when compared to the knee joint arthritis (zymosan-treated) control group (Figure 5(b)). The knee joint arthritis (zymosan-treated) control group showed elevated influx levels of mast cells to the knee joint tissues (Figure 6) with a high mean total number of mast cell count of 30.80 ± 3.06 (Figure 6). However, the aspirin (100 mg·kg^−1^)-treated groups decreased the proliferation of mast cells in the knee joint tissues (Figure 5(c)) and significantly (*P* < 0.05) reduced the mean total number of mast cell count to 15.20 ± 1.66 when compared to the arthritic (zymosan-induced) control group (Figure 6). Similarly, the PLE-treated (100–600 mg·kg^−1^) group showed a decreased influx of mast cells to the knee joint tissues (Figures 5(d)–5(f)) with a significant (*P* < 0.05) reduction in the mean total number of mast cell count to 21.20 ± 3.34, 18.20 ± 2.85, and 17.00 ± 2.00 at 100, 300, and 600 mg·kg^−1^ when compared to the zymosan-treated (acute knee joint inflamed) control group, respectively (Figure 6).

#### 4.1.4. Histamine-Induced Paw Oedema in Rats

**Figure 7:**
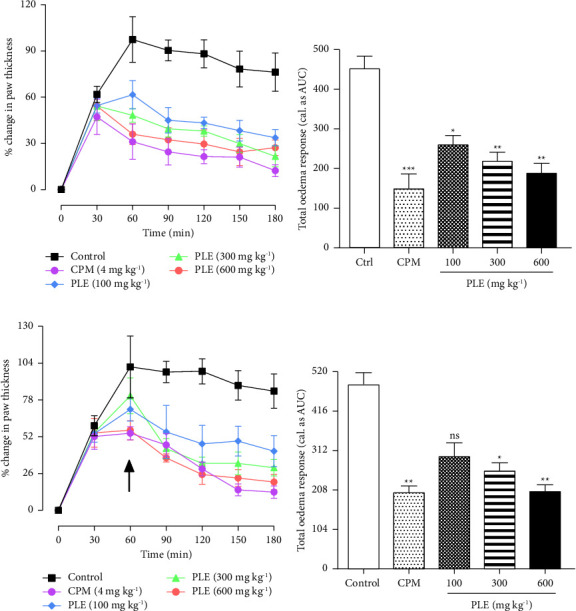
Effect of PLE on histamine-induced paw oedema in rats. Either chlorpheniramine (4 mg·kg^−1^, p.o) or PLE (100–600 mg·kg^−1^, p.o) was administered prophylactically (a, b) prior to oedema induction or therapeutically (c, d). Total paw oedema was determined as AUC (b, d), and data were presented as mean ± S.E.M. ^*∗*^*P* < 0.05; ^*∗∗*^*P* < 0.01; ^*∗∗∗*^*P* < 0.001 compared to the inflamed control response (two-way ANOVA followed by Dunnet's *post hoc* test). The arrow denotes the time of drug administration. *ns* = nonsignificant; CPM = chlorpheniramine.

From the study, it was revealed that PLE (100–600 mg·kg^−1^, p.o) administered before oedema induction caused the mean maximal inflamed paw oedema formed at 60 min to be suppressed significantly to 61.53 ± 9.17%, 54.21 ± 9.38%, and 54.22 ± 9.37% at 100, 300, and 600 mg·kg^−1^ when compared to the inflamed control maximal response of 97.38 ± 14.87%, respectively (Figure 7(a)). Furthermore, the total inflamed paw oedema attained over 180 min (cal. as the area under the time-course curve, AUC) was reduced significantly by 42.88%, 51.44%, and 58.12% at the same doses relative to the inflamed control response, respectively (Figure 7(b)). In the therapeutic study, PLE (100–600 mg·kg^−1^, p.o) reduced the mean maximal oedema formed at 60 min to 80.89 ± 12.60% and 56.59 ± 6.77% at 300 and 600 mg·kg^−1^ when compared to the inflamed control response (101.38 ± 21.87%), respectively (Figure 7(c)). Similarly, the total paw oedema attained over 180 min (cal. as the area under the time-course curve, AUC) was also reduced by 46.46% and 57.62% at the same doses relative to the inflamed control response, respectively (Figure 7(d)).

## 5. Discussion

Toxicity screening of medicinal plants using animal models is essential to validate their use in regular therapy [19] because the effects observed in animals as a result of exposure to chemical substances can be related to humans [20]. Based on this assertion, the acute toxicity profile of *Persicaria lanigera* was assessed to confirm its safety using acute toxicity studies in rats. The animals used in the study survived in all doses after oral administration of PLE at the end of the observation period. PLE-treated (100–5000 mg·kg^−1^, p.o) animals showed no noticeable behavioural, physiological, or clinical alterations or any toxic effect, and there was no death. However, some mild, short-lived signs such as defaecation and urination were observed in rats at doses of 3000 mg·kg^−1^ and 5000 mg·kg^−1^, and nevertheless, these signs gradually diminished within a period of 24 h after PLE administration. From the study, the data obtained revealed that the LD_50_ of PLE could be estimated to be above 5000 mg·kg^−1^, and therefore, it is important to establish that PLE is relatively safe for its usage in traditional medicine, which conforms to the scale based on the Hodge and Steiner toxicity scale [21]. For this reason, the confirmation of the *Persicaria lanigera* safety profile further supported the assessment of its anti-inflammatory activity on inflammatory models.

To investigate inflammation, several experimental models are used to evaluate inflammatory responses. The methods used to assess a substance's anti-inflammatory effectiveness are often conducted on test animals, including other biochemical models. The most common test in the quest for new, complementary, and alternative anti-inflammatory medications focuses primarily on evaluating an agent's ability to reduce oedema that is brought on by the injection of phlogistic substances in animals [22]. Thus, to investigate the anti-inflammatory effects of PLE on acute inflammation, carrageenan-induced paw oedema, histamine-induced paw oedema, and zymosan-induced acute knee joint arthritis in rats were employed.

Carrageenan-induced paw oedema is a typical acute inflammatory model used in animal studies to evaluate the anti-inflammatory activity of a test compound. It is a sensitive and reproducible model used to assess new compounds. The model is useful for discovering orally active anti-inflammatory compounds that act through acute inflammatory mediators [23]. It has also been observed that lipoxygenase (LOX) and cyclooxygenase (COX) inhibitors are useful in combating carrageenan-induced paw oedema [24].

The injection of carrageenan causes a biphasic induction of oedema over time, and the anti-inflammatory effect is normally determined after 6 h due to the depletion of an inflammatory cofactor, kininogen, after this period [25]. In the early phase (0–2 h after carrageenan injection), proinflammatory mediators such as histamine, bradykinin, and serotonin (5-HT) are involved. The late phase, which occurs after 2 h, is mediated by the release of prostaglandins, nitric oxide, TNF-*α*, free radicals, oxygen species, and leukotrienes [26]. In the study, treatment with PLE showed significant inhibition of oedema both in the prophylactic (preemptive, 2 h before carrageenan injection) and curative studies (2 h after carrageenan injection). PLE was a potent inhibitor of the initial phase, which suggests that the anti-inflammatory effect of PLE could be attributed to the inhibition of proinflammatory mediators such as histamine, bradykinin, and 5-HT.

The extract also significantly inhibited the late phase, suggesting its high inhibitory effects on the metabolic arachidonic acid pathway. The potential action of PLE in the late phase also suggests that other proinflammatory mediators, such as nitric oxide and leukotrienes, were inhibited. Furthermore, a proinflammatory mediator such as histamine, which plays a major role in acute inflammation [27], was significantly inhibited by PLE in the histamine-induced paw oedema model via downregulation of the synthesis as well as its effects.

The basic understanding of inflammatory joint illnesses and the creation of medications with efficient anti-inflammatory and antiarthritic activities have both benefited from the use of animal models, despite their many limitations.

Zymosan-induced acute knee joint arthritis is widely used to evaluate the anti-inflammatory activities of various compounds [28]. During inflammatory responses as a result of zymosan-induced inflammation, the actions of phagocytosis, cell migration, and the synthesis of proinflammatory mediators can be studied [18]. Zymosan has been shown to stimulate phagocytic cells, which increases the quantity of lysosomal enzymes secreted, boosts the release of proinflammatory cytokines including TNF-*α* and IL-6, and increases the leukotriene synthesis of monocytes, chemokines such as chemokine-C-X-C motif (CXCL-1), matrix metalloproteinase-9, and monocyte chemoattractant protein (MCP-1) [29].

When zymosan is injected into joints, it induces biphasic arthritis in rodents. The early stage is marked by increased lymphocyte and macrophage production, whereas the late stage is mediated by increased vascular permeability, oedema, leukocyte infiltration, and the influx of neutrophils [30]. Previous reports have also shown that proinflammatory cytokines such as NF-*κ*B, TNF-*α*, IL-1*β*, IL-6, and ROS [31] are involved in zymosan-induced acute knee joint arthritis in rats.

This study revealed that oral administration of PLE before the injury dramatically reduced the thickness of the knee joints in the right limbs of rats. As a result, oedema caused by zymosan injection into the articular cavity was significantly reduced, which in turn led to the inhibition of proinflammatory mediators that are involved in acute knee joint inflammation. This antiarthritic action of PLE may be explained by the suppression of several cytokines, enzymes, ROS, and other proinflammatory mediators involved in arthritic inflammation. Therefore, it can be said that PLE has antiarthritic action, consistent with past research showing that plants can treat inflammatory diseases such as arthritis by reducing inflammation [32].

It has been reported that intra-articular injection of zymosan stimulates massive migration of leukocytes, especially neutrophils, into the synovial tissue and fluids of inflamed joints [31]. Neutrophils have been shown to play a key role in the pathogenesis of joint arthritis and promote cartilage damage as well as bone resorption in the joints via the production of ROS in association with other proteolytic enzymes [33]. Neutrophils are also reported to induce the release of proinflammatory cytokines such as TNF-*α*, IL-1*β*, IL-6, and chemokines including CXCL-1 that cause bone and cartilage damage [31]. Therefore, inhibiting neutrophil infiltration or activation is a crucial treatment option for arthritis.

In the study, treatment with PLE significantly attenuated the influx of neutrophils and the migration of leukocytes into the synovial cavity of the knee joint and thus potentially inhibited the production of proinflammatory mediators and other proinflammatory cytokines such as TNF-*α*, IL-1*β*, and IL-6 that could cause cartilage and bone damage. The reduction of neutrophil and leukocyte levels by the extract in the knee cavity and fluids of the inflamed joints contributed to the management of the arthritic condition. This is in consonance with the literature which previously reported that established attenuation of increased neutrophil infiltration is relevant in the management of arthritis [34].

According to Babu et al., histopathological changes in an arthritic knee joint are characterized by severe cell infiltration, loss of synovial space, cartilage and bone erosion, and distortion of the synovial membrane lining [35]. Hence, these pathological alterations are known to be clinical features of degenerative joint disorders. In addition, it has also been reported that bone and cartilage erosion in the arthritic knee joint is mediated by proinflammatory mediators and cytokines [36]. In the study, PLE showed a significant improvement in the histological changes in the knee joint of rats caused by the bone and cartilage tissues, synovial membrane lining, and synovial space. This was evident in its inhibitory effects on cartilage and bone degradation, decreased synovial space, increased inflammatory cell infiltration, and proinflammatory cytokines as well as mediators implicated in knee joint arthritis. Hence, bone remodeling could be upregulated to maintain bone integrity, and therefore, this agrees with earlier literature that reported the established ability of plant extracts to maintain the histopathological architecture of the bone and cartilage during arthritic inflammation [35].

Chan et al. reported that mast cells are implicated in the pathogenesis of osteoarthritis and their levels are proliferated in the osteoarthritic bone [37]. They are also said to be present in the synovium and synovial fluids of patients with knee osteoarthritis [38]. Mast cells, upon activation, release a series of proinflammatory mediators such as histamine, proinflammatory lipids (prostaglandins), chemokines, and cytokines including TNF-*α* and and IL-6 [39] that regulate bone metabolism potentiating bone resorption [40]. Based on this assertion, the antiproliferative effect of PLE on mast cell proliferation or activation in this study was very significant. PLE remarkably reduced the mast cell levels in the synovium and synovial fluid of the knee joint cavity and consequently inhibited the proinflammatory cytokines and mediators that could be released to modulate bone metabolism, leading to increased bone resorption to cause cartilage and bone erosion. This aligns with the literature earlier reported that established the inhibitory effects of medicinal plants to suppress mast cell activation or proliferation in bone tissue as a key mechanism in arthritic therapy [37].

## 6. Conclusion

Putting all the above data from the study together, it can be concluded that the aqueous ethanolic leaf extract of *Persicaria lanigera* is a potent anti-inflammatory agent and could be effective against both acute and chronic inflammation. This study has revealed for the first time that the *Persicaria lanigera* leaf extract inhibits oedema, joint inflammation, and colonic damage through its inhibitory effects on histamine, mast cells, and inflammatory cells such as neutrophils.

## Figures and Tables

**Table 1:** Observations in the acute toxicity study postoral administration of PLE in rats.

Toxicity signs	Control	100	300	1000	3000	5000
Mortality	NØ	NØ	NØ	NØ	NØ	NØ
Latency (h)	—	—	—	—	—	—
Tremor	0	0	0	0	0	0
Excitement	0	0	0	0	0	0
Convulsion	0	0	0	0	0	0
Respiratory abnormality	0	0	0	0	0	0
Aggression	0	0	0	0	0	0
Agitation	0	0	0	0	0	0
Unusual locomotion	0	0	0	0	0	0
Ataxia	0	0	0	0	0	0
Sedation	0	0	0	0	0	0
Salivation	0	0	0	0	0	0
Defecation	0	0	0	0	1	1
Urination	0	0	0	0	1	1
Diarrhea	0	0	0	0	0	0
Reactivity to touch	0	0	0	0	0	0

0 = normal; 1 = mildly impaired; 2 = moderately impaired; 3 = severely impaired; NØ = no death.

## Data Availability

The data used to support this study are available upon request.
